# Subsequent Fertility in Women Treated for Caesarean Scar Pregnancy With Hysteroscopy: A 5-Year Follow-Up Descriptive Study in a Tertiary Hospital

**DOI:** 10.3389/fendo.2021.659647

**Published:** 2021-05-10

**Authors:** Xinyi Sun, Yang Liu, Yunhui Tang, Hongying Yu, Min Zhao, Qi Chen

**Affiliations:** ^1^ Department of Obstetrics and Gynaecology, The University of Auckland, Auckland, New Zealand; ^2^ School of Medicine, Nanjing Medical University, Nanjing, China; ^3^ Department of Family Planning, The Hospital of Obstetrics and Gynaecology, Fudan University, Shanghai, China; ^4^ Department of Ultrasound, The Affiliated Wuxi Maternity and Child Health Care Hospital of Nanjing Medical University, Wuxi, China; ^5^ Department of Gynaecology, The Affiliated Wuxi Maternity and Child Health Care Hospital of Nanjing Medical University, Wuxi, China

**Keywords:** caesarean scar pregnancy, subsequent pregnancy, subsequent fertility, complications of pregnancy, follow-up, hysteroscopy

## Abstract

**Objective:**

The outcomes of subsequent pregnancies and fertility in women with a history of caesarean scar pregnancy have not been well described. In this study, we followed up 149 women with a history of caesarean scar pregnancy and analysed the effect on their fertility.

**Methods:**

149 women with a history of caesarean scar pregnancy were followed up for five years. Of them, 53 women had unprotected sexual intercourse attempting to become pregnant again. Data including clinical parameters and treatment options at the time of diagnosis of caesarean scar pregnancy, and the outcomes in subsequent pregnancy were collected. In addition, a questionnaire about the menstrual cycle after treatment was voluntarily completed by these women.

**Results:**

Of the 53 women, 46 (84%) women had a subsequent pregnancy, while seven (14%) women did not. There was no association between the clinical parameters in previous caesarean scar pregnancy or treatment and future fertility. From the questionnaire, there was no difference seen in the length of the menstrual cycle and menses between the two groups. However, a higher number of women with light menstrual bleeding were seen in women without a subsequent pregnancy (67%), compared with women who did (28%). In addition, six women (13%) who had a subsequent pregnancy experienced foetus death in the first trimester.

**Conclusion:**

We reported that 14% of women with a history of cesarean scar pregnancy did not have a subsequent pregnancy, after unprotected sexual intercourse for more than two years. Light menstrual bleeding after treatment may be associated with this adverse effect. Our findings need to be further investigated with large sample size.

## Introduction

The incidence of caesarean scar pregnancy is relatively low, with an estimated 0.04% to 0.05% of pregnancies worldwide (1). However, with an increase in the caesarean section rate worldwide, in particularly in China, the incidence of caesarean scar pregnancy has significantly increased in last decade. Given the risk of life-threatening complications, management of caesarean scar pregnancy is becoming another challenge for gynaecologists. Although five treatment options (transvaginal resection; laparoscopy; uterine artery embolization combined with dilatation, curettage, and hysteroscopy; uterine artery embolization in combination with dilatation and curettage; and hysteroscopy) are recommended (2–4), to date there is still no agreement on the most optimal management of caesarean scar pregnancy. This is because of the limited number of clinical studies (including clinical trials) with a large enough sample size (5, 6) and this consequently results in the majority of studies on caesarean scar pregnancy being reported in the literature as case series. Therefore, hysteroscopy resection is the most common option for cesarean scar pregnancy treatment in China, including in our hospital, although this is a less commonly used treatment option, especially in the United States (2, 7).

Currently, data on subsequent outcomes in women with a history of caesarean scar pregnancy have not been well described. Although a relatively good outcome with a live birth in subsequent pregnancies and a lower recurrence rate of caesarean scar pregnancy has been reported (8–11), our recent study suggested that there may be a potential risk for developing complicated pregnancies, such as gestational diabetes mellitus (GDM) (11). Because of the potential risk of developing recurrent caesarean scar pregnancies, most women with a history of caesarean scar pregnancy did not attempt to have a subsequent pregnancy. This consequently results in a limitation to investigate the adverse effects of caesarean scar pregnancy. For this reason, to date there is a lack of data on subsequent fertility in women with a history of caesarean scar pregnancy after receiving initial treatment.

Therefore, we conducted a five-year follow-up series study with a relatively large sample size to investigate future reproductive ability in women with a history of caesarean scar pregnancy mainly treated with hysteroscopy in a tertiary hospital.

## Materials and Methods

This follow-up study received approval by the Ethics Committee of Wuxi Maternity and Child Health Hospital, Nanjing Medical University, China.

### Study *P*opulation

One hundred forty-nine (64%) women from a total of 232 women who were diagnosed with caesarean scar pregnancy in Maternity and Child Health Hospital, Nanjing Medical University, China between January 2016 and December 2018, were followed up until December 2020. Of 149 women, 111 were included in our previous study (11). Data on maternal age, parity, gravida, gestational sac age at diagnosis, the size of sac, the option(s) of treatment, the amount of bleeding during the initial treatment and the serum levels of β-hCG at diagnosis and after initial treatment were collected from the hospital electronic database. In addition, all these women (n=149) were asked to voluntarily complete a questionnaire at the time of collection (**Supplementary File 1**). Questionnaire included protected or unprotected sexual intercourse, the average length of menstrual cycles and menses, and menstrual bleeding condition after caesarean scar pregnancy treatment. A self-reported light menstrual bleeding during menstrual menses was defined as a comparison of the amount of menstrual bleeding before and after caesarean scar pregnancy treatment. Ultrasound data after an initial treatment of previous caesarean scar pregnancy on these women were also collected from the hospital electronic database.

The diagnosis of caesarean scar pregnancy was based on findings from the transvaginal ultrasound image including the presence of a gestational sac in the area of the scar using VolusionE8 model, in addition to a history of a prior caesarean section and a positive pregnancy test. Infertility was defined as a failure to achieve a clinical pregnancy after 12 months or more of regular unprotected sexual intercourse recommended by World Health Organisation (WHO).

### Statistical Analysis

Data on age, gestational age, sac size and levels of β-hCG, length of menstrual cycle or menses were presented as mean and standard deviation (SD). The options of treatment and menstrual bleeding were presented as a percentage. Due to the small sample size, only descriptive statistics were performed for this study.

## Results

**Figure 1:**
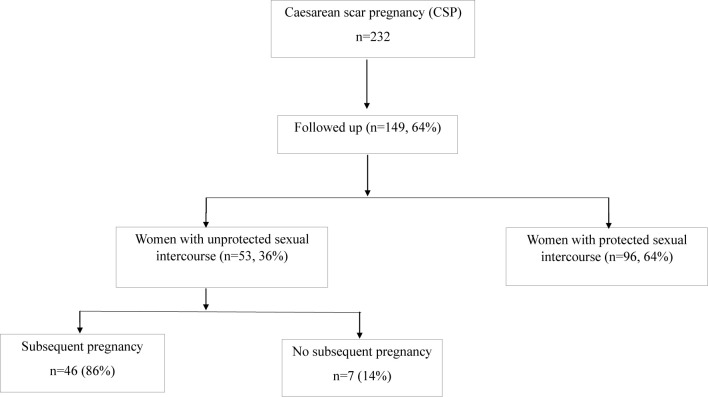
Flowchart of study population.

Over the period of study, 149 women with a history of caesarean scar pregnancy were followed up. Of them, there were 96 (64%) women had protected sexual intercourse to avoid further pregnancy after an initial treatment of caesarean scar pregnancy, as they were fearful of another caesarean scar pregnancy. Fifty-three women who attempted to become pregnancy again had unprotected sexual intercourse after an initial treatment of caesarean scar pregnancy. In these 53 women, 46 (86%) women had a subsequent pregnancy (one woman had two pregnancies) during the study period, and two (4.3%, 2 out of 46 women with a subsequent pregnancy) of them previously had two or three recurrent caesarean scar pregnancies. Due to the outcomes of subsequent pregnancy may be different in women with multiple scar caesarean pregnancies, these two cases were then excluded for further analysis. While seven (14%) women did not have a subsequent pregnancy after at least 2 years of treatment of the caesarean scar pregnancy (**Figure 1**). In addition, we also found that six women (13.6%, 6 out of 44 women with a subsequent pregnancy) who had a subsequent pregnancy experienced a missed miscarriage in the first trimester.

The clinical characteristics in these women with a history of caesarean scar pregnancy who had or did not have a subsequent pregnancy after unprotected sexual intercourse are summarised in **Table 1**. There was no difference in the maternal age at onset of caesarean scar pregnancy, gestational age, size of sac, serum levels of β-hCG at diagnosis, and the numbers of surgical termination between the two groups. In addition, there was also no difference in the option of initial treatment between the two groups.

**Table 1:** Clinical characteristics in women with a history of caesarean scar pregnancy who had or did not have a subsequent pregnancy.

	Women with a subsequent pregnancy (n = 44)*	Women without a subsequent pregnancy (n = 7)
Age at previous CSP (years, mean/SD)	31.4 ± 4.1	35.1 ± 5.9
Gestational age (days, mean/SD)	50.5 ± 13.6	51.7 ± 13.3
Sac size (mm, mean/SD)	29.4 ± 16.1*13.2 ± 6.5	27 ± 23.1*11.7 ± 11.7
β-hCG at diagnosis (IU/L, median/range)	25887 (442.9 to 225032)	17337 (1386 to 146650)
Previous termination (n, mean)	2	2
**Previous treatment** uterine curettage with or without		
TX (n, %)	8 (18%)	2 (29%)
Hysteroscopy (n, %)	29 (66%)	5 (71%)
Laparoscopy (n, %)	2 (4.5%)	0 (0%)
Transvaginal resection combined by hysteroscopy (n, %)	5 (11.5%)	0 (0%)

*Two cases with multiple scar caesarean pregnancies were excluded.

To understand whether developing caesarean scar pregnancy could impact fertility later, women who were followed up were asked to voluntarily complete a questionnaire about the menstrual cycle after treatment of caesarean scar pregnancy. From the questionnaire, there was no difference seen in the days of the menstrual cycle restarting, the length of the menstrual cycle and menses between the two groups (**Table 2**). However, we found that a higher number of women without a subsequent pregnancy had light menstrual bleeding (66.7%), compared to women with a subsequent pregnancy (28%), or women with protected sexual intercourse (22.9%) after initial treatment of caesarean scar pregnancy (**Table 2**). In addition, six women (13.6%, 6 out of 44) who had a subsequent pregnancy and one (14%, one out of seven) woman who did not have a subsequent pregnancy, had caesarean scar niche after the initial treatment of caesarean scar pregnancy.

**Table 2:** Information on menstrual cycle in women with or without a subsequent pregnancy after an initial treatment of caesarean scar pregnancy.

	Women with a subsequent pregnancy (n = 44)*	Women without a subsequent pregnancy (n = 7)	Women with protected sexual intercourse (n = 96)
menstrual cycle back (days, mean/SD)	30.4 ± 2	30.0 ± 0	32.2 ± 7.6
Length of cycle (days, mean/SD)	30.4 ± 2	30.0 ± 0	32.2 ± 7.6
Length of menses (days, mean/SD)	5.2 ± 1.5	5.7 ± 1.0	5.6 ± 1.89
Menstrual bleeding (number, %)**			
Light	9 (28%)	4 (66.7%)	21 (22.9%)
Same	23 (72%)	2 (33.4%)	70 (76%)
Heavy	0	0	1 (1.1%)

*Two cases with multiple scar caesarean pregnancies were excluded.

**Data on menstrual bleeding were not available in 12 cases or one case or four cases from women with a subsequent pregnancy or without a subsequent pregnancy or women with protected sexual intercourse, respectively.

## Discussion

In this follow-up series study, we demonstrated that 64% (96 out of 149) of women with a history of caesarean scar pregnancy avoided having a subsequent pregnancy in our population. In women with unprotected sexual intercourse who attempted pregnancy again after an initial treatment of caesarean scar pregnancy (n=53), 46 (86%) women had a subsequent pregnancy, while seven (14%) women did not. The numbers of women with the light menstrual period after an initial treatment of caesarean scar pregnancy were higher in women without a subsequent pregnancy (66%, 4 out of 6), compared to women with a subsequent pregnancy (28%, 9 out of 32).

To date there were very few studies investigating the association of caesarean scar pregnancy with subsequent fertility in the literature. This could be because that the majority of women with a history of caesarean scar pregnancy are afraid of developing recurrent caesarean scar pregnancy, resulting in women not attempting to become pregnant again, even the rate of recurrent caesarean scar pregnancy is relatively low (8–11). A six year follow-up study led by Nagi reported that three (12.5%) women did not become pregnant, in a total of 24 women treated for caesarean scar pregnancy who attempted to conceive (8). Another study reported 13 (16.5%) women treated for caesarean scar pregnancy did not achieve a subsequent pregnant, in a total of 79 women who tried to conceive (12). Recently another five year follow-up study also reported that six pregnancies occurred in 10 women with a history of caesarean scar pregnancy who attempted to further conceive after an initial treatment, suggesting four women (40%) were not pregnant after more than 12 months (9). A most recent study also reported the reduced pregnancy and live birth rate after IVF in women with a history of scar caesarean pregnancy (13). In our current study, we found that seven (14%) women with a history of caesarean scar pregnancy did not have a subsequent pregnancy who had unprotected sexual intercourse, which was similar to Nagi’s study (8). The difference in the rate of non-subsequent pregnancy between these studies including our one could be due to the sample size, as one of the factors. However, the potential reasons for those women who were not successful in having a subsequent pregnant in these studies were not further investigated (8, 9, 12).

There are many factors contributing to subsequent fertility. The menstrual cycle could be changed after termination (14), which could be because of the damage of the endometrium or the delay of recovery of the endometrium or changes in hormones. Irregular menstrual cycle includes changes in the length of cycle or menses and menstrual bleeding. Menstrual bleeding for less than two days, or a light menstrual period is considered to be scanty. Scanty bleeding is usually an indication that the lining of the uterus is not as lush or thick. Light menstrual periods are also a big sign that the uterine lining is not getting the circulation it needs to really build up to the thickness for an embryo to implant (15, 16), and women with light menstrual bleeding are associated with a poor conception outcome (17). In our current study, we found that there was no difference in the length of the menstrual cycle and menses between women with a history of caesarean scar pregnancy who had or did not have a subsequent pregnancy after unprotected sexual intercourse. However, 66.7% of these women without a subsequent pregnancy had a light menstrual period, which was much higher than that in women with a subsequent pregnancy (28%), as well as much higher than women with protected sexual intercourse (23%) after an initial treatment of caesarean scar pregnancy. There is currently no evidence indicating treatment options of caesarean scar pregnancy resulting in light menstrual bleeding¸ although a recent follow-up study (up to 57 months) reported that 60% of women with a history of caesarean scar pregnancy had reduced menstrual blood volume or amenorrhea after an initial treatment with uterine artery embolization (18). Changes in the uterine tree could cause a dysfunctional menstrual bleeding (19). In addition to the menstrual cycle, we also analysed the follow-up ultrasound check. However, abnormal ultrasound findings on the uterus and ovaries in these women within 5 months after an initial treatment of caesarean scar pregnancy were not seen. Unfortunately, ultrasound data on long term follow-up was not available. Therefore, future studies are required to follow up the abnormalities of uterus and hormone levels in women with a history of caesarean scar pregnancy after an initial treatment for long time. In addition, Asherman’s syndrome from dilation and curettage (D&C) or a caesarean section (20) is one of the causes of light menstrual bleeding (21).

Caesarean scar niche has been suggested to underlie some cases of subfertility (13, 22). In our current study we found that six women who had a subsequent pregnancy had caesarean scar niche (13%) and one woman who did not have a subsequent pregnancy had caesarean scar niche (14%) after initial treatment of caesarean scar pregnancy. It seems the presence of caesarean scar niche after initial treatment does not associate with subsequent fertility, but this needs to be further investigated with a large sample size study (13). In addition, only one woman without a subsequent pregnancy had a fibrosis. We do not know yet whether this was the underlying risk factor for infertility.

Currently expectant, medical and surgical treatment are recommended by RCOG/AEPU Green-top Guideline for selective abortion (4). Although non-surgical management of caesarean scar pregnancy is a more common option in western countries such as the United States of American (2, 7), in our hospital (maybe also in most hospitals in China), hysteroscopy is the most common option for cesarean scar pregnancy treatment. A recent study reported that high intensity focused ultrasound (HIFU) followed by ultrasound guided dilation and curettage (D&C) treatment in previous caesarean scar pregnancy seems to have a lower risk in recurrent caesarean scar pregnancy and other complications in subsequent pregnancies (10). However, this finding was not seen in other studies (7, 9, 11). In our current study, we also found no difference in the treatment option of caesarean scar pregnancy between women with and without a subsequent pregnancy, suggesting previous treatment options of caesarean scar pregnancy may not have any effect on subsequent fertility of women. Previous caesarean section may be a risk for causing future infertility (23); however, in our current study, we also did not see this potential association. Interestingly, in our current study, we also found that six women (13%) with a subsequent pregnancy experienced a missed miscarriage in the first trimester of pregnancy, which is similar with the overall miscarriage rate worldwide (24–26). Because all our women previously had at least one live birth without complications and did not previously experience miscarriage, we do not know the exact cause(s) of missed miscarriage shown in the first trimester in these women, in relation to either previous scar caesarean pregnancy or the options of previous treatment. To date, there is no agreement on the most optimal management of caesarean scar pregnancy, we are currently performing a randomised clinical trial (reference number KYY2020-185) to investigate whether the treatment option(s) of caesarean scar pregnancy is associated with complication(s) of subsequent pregnancy, and recurrent caesarean scar pregnancy, and subsequent infertility.

The relatively small sample size of women who did not become pregnant again is one of the main limitations of our study. But the sample size on women who attempted to become pregnant again was relatively large (36%). In addition, many factors could contribute to infertility, while in this study we only analysed the changes in the menstrual cycle after an initial treatment of caesarean scar pregnancy. Future studies with confounders are required to confirm our findings. Data on the menstrual cycle after an initial treatment of caesarean scar pregnancy were self-reported, which may cause a bias. We also do not know the fertility ability in those women who had protected sexual intercourse after an initial treatment of caesarean scar pregnancy. Lastly, an analysis of only 64% of the originally treated group may introduce bias as well. Due to the majority of cases reported in this study were previously treated with hysteroscopy, our data may not present all the scar caesarean pregnancy.

A broad spectrum of options on the treatment of cesarean scar pregnancy based on the clinical presentations including patients’ desires for further fertility results in a real challenge for gynaecologists. In conclusion, in our descriptive study, we found 14% of women with a history of caesarean scar pregnancy who had unprotected sexual intercourse after an initial treatment did not have a subsequent pregnancy. The changes in menstrual bleeding after treatment may be one of the reasons for this adverse effect. Our findings need to be investigated in future with large sample size.

## Data Availability Statement

The original contributions presented in the study are included in the article/**Supplementary Material**. Further inquiries can be directed to the corresponding author.

## Ethics Statement

The studies involving human participants were reviewed and approved by Ethics Committee of Wuxi Maternity and Child Health Hospital, Nanjing Medical University. Written informed consent for participation was not required for this study in accordance with the national legislation and the institutional requirements.

## Author Contributions

XS and YL collected the data reported in this work. YT, HY, MZ, and QC contributed to the conception and design of this study. XS contributed to data analysis. MZ and QC wrote the manuscript draft. All authors contributed to the article and approved the submitted version.

## Funding

This study received support from the Outstanding Talent Project of Wuxi Health and Family Planning Commission of China (ZDRC023 to MZ) and the Medical Innovations Project of Shanghai Science and Technology Committee of China (20Y11907400 to YT).

## Conflict of Interest

The authors declare that the research was conducted in the absence of any commercial or financial relationships that could be construed as a potential conflict of interest.

## Supplementary Material

The Supplementary Material for this article can be found online at: https://www.frontiersin.org/articles/10.3389/fendo.2021.659647/full#supplementary-material
