# Supplementary material for: Subsequent Fertility in Women Treated for Caesarean Scar Pregnancy With Hysteroscopy: A 5-Year Follow-Up Descriptive Study in a Tertiary Hospital
Source: Front Endocrinol (Lausanne). 2021 May 10;12:659647. doi: 10.3389/fendo.2021.659647 (PMC8142377; doi:10.3389/fendo.2021.659647)
Supplement: Supplementary file 1 [file DataSheet_1.doc]

**Questionnaire**

**Dear Madam**

We are undertaking a study to investigate whether caesarean scar pregnancy (CSP) including the option of treatment is associated with infertility. We would like to warmly invite you to participate this survey and answer all the questions listed below, as many as you can.

All your information is strictly confidential and will be only used for this study. Only research team members are able to access to the data and no other public party will be involved in this study.

We much appreciate your time on completion of this survey. If you have any question, please contact to Dr. Min Zhao by 86-1386168860 for assistant. This study was approved by the Ethics board of Wuxi Maternity and Child Health Hospital, Nanjing Medical University, China.

**Questions**

1. Did you have a subsequent pregnancy after treatment?
2. Did you attempt to protect for getting subsequent pregnancy after treatment?
3. If yes to question 1, how long were you pregnant after treatment?
4. If yes to question 1, did you keep the pregnancy?
5. If yes to above question, what gestational age did you give a birth?
6. If yes to above question, did you have any complications of pregnancy?
7. How long did the menstrual cycle restart after treatment?
8. The average length of menstrual cycles (days) after treatment:
9. The average length of menses (days) after treatment:
10. The amount of blood loss during your menses after treatment, comparing with before treatment (please circle one):

Less Same More

1. Do you have any dysmenorrhea after treatment?
2. Do you have any chronic gynaecological diseases or other endocrinological disorders, such as uterine fibroids, endometriosis, PCOS, medication for menstruation, irregular menstruation?

Thanks again for your time.

Research Team: Drs. Xinyi Sun, Yang Liu, Yunhui Tang, Hongying Yu, Min Zhao, Qi Chen
